# The Treatment of Turbinal Hypertrophy

**Published:** 1901-06-29

**Authors:** 


### THE TREATMENT OF TURBINAL HYPERTROPHY.

Various as are the methods which have from time to time been adopted for the reduction of hypertrophy of the mucous membrane covering the turbinal bones, it cannot be said that any of them have received universal acceptance; in fact, some of the most effectual of them, which take the form of turbinectomy in one form or another, can hardly be spoken of as methods for reducing turbinal hypertrophies, seeing that they go rather on the good old scriptural advice to cut off the organ that offends. In this disease the tissues most directly involved are the mucous membrane covering the inferior and middle turbinals and the underlying erectile and vascular tissue, which, as the result of the hypertrophic changes which it has undergone, is mainly responsible for the diminished calibre of the nares and the consequent difficulty in nasal respiration; and whether this hypertrophy is to be sliced off, or cauterised, or scarified, or what not, in all the aim is to open up the air passage. Undoubtedly turbinectomy or turbinotomy will do this, and if all our patients were chronics suffering from advanced conditions there would be little to say against these proceedings. But with the advance of specialism the specialist finds that an increasing proportion of his cases are of the milder sort, and in regard to this disease instead of the rigid and partly bony hypertrophy which nothing but strong measures can relieve, it is found that not a few patients are suffering from a soft swelling of the soft tissues only. In view of this condition of affairs, Dr. Goldstein,[1] of St. Louis, advises the employment of submucous cauterisation, a process in the performance of which, however, he has met with some difficulties, chiefly in getting the caustic to follow the tract of the preliminary puncture. With the object of obviating these troubles he recommends the use of a special trocar and canula armed with a sharp well-fitting obturator, and fitted with a ring limiting the puncture to any required depth. The area to be operated on is cocainised with a 4 per cent. solution. The depth of the hypertrophy is estimated and the ring adjusted. The sharp trocar is introduced, generally about a quarter of an inch from the muco-cutaneous junction, and plunged into the hypertrophied mass in a direction parallel to the turbinate bone, "and hugging the surface of the bone" until the ring guard is reached. The trocar is then removed, and the probe on which a bead of chromic acid has been fused is then introduced to the area to be cauterised. The probe also carries a ring guard so that it may project about a quarter of an inch beyond the canula. The canula, with the probe still projecting, is now gradually withdrawn, by which proceeding the entire turbinal area to be cauterised is brought into contact with the chromic acid. The operation ends with the application of a campho-menthol spray and a tampon. The results are said to be satisfactory and permanent.

[1] The Laryngoscope, May, 1901.

